# Supplementary material for: Detecting adherence to the recommended childhood vaccination schedule from user-generated content in a US parenting forum
Source: PLoS Comput Biol. 2021 Apr 26;17(4):e1008919. doi: 10.1371/journal.pcbi.1008919 (PMC8075195; doi:10.1371/journal.pcbi.1008919)
Supplement: S1 Appendix — This document provides supplementary information regarding: data collection and structure of the dataset (section 1), extraction pipelines (section 2), geolocation of users (section 3) and construction of the interaction network (section 4). (PDF) [file pcbi.1008919.s001.pdf]

Detecting adherence to the recommended childhood vaccination schedule from user-generated content in a US parenting forum

Lorenzo Betti[1*], Gianmarco De Francisci Morales[1], Laetitia Gauvin[1], Kyriaki Kalimeri[1], Yelena Mejova[1], Daniela Paolotti[1], Michele Starnini[1]

1 ISI Foundation, Turin, Italy

* lorenzo.betti@isi.it

S1 Appendix

1 Data collection and structure of the dataset

The forum hosted by the website BabyCenter.com is devoted to parental support which consider all aspects of parenting. In order to retrieve vaccine-related content, we queried the site search function with the word '*vaccine*' and the response consisted on a series of posts, that we assumed relevant to vaccination. We used the technique of web-scraping to collect all the addresses of the retrieved posts. In a second step, we collected the source code of the webpages containing each post along with the corresponding comments. After that, we extracted all the nick names of users and corresponding addresses, and then we scraped all the publicly accessible profile pages. Finally, we extracted all the relevant information from the data collected. In particular:

- for posts : title of the post, date of publication of the post, author of the post, text content of the post, the group where the post were submitted, identifier of the post;
- for comments : date of publication of the comment, identifier of the post under which the comment were submitted, author of the comment, text content of the comment, identifier of the comment;
- from user profile pages : user name, self-reported geolocation, list of groups joined

2 Extraction pipelines

2.1 Vaccination schedule extraction pipeline

In the following, we describe the extraction pipeline developed to retrieve and classify schedule-related comments into two classes: '*recommended*' or '*alternative*'. Let us consider these two sentences containing two keywords relevant respect to vaccination scheduling ('*schedule*' and '*delay*'):

"*I am on a regular vaccination schedule*"
"*My friend suggests to delay vaccines*".

In the first, the author of the comment ('*I*') talks about following the recommended schedule, while in the second the author does not refer to the vaccination schedule adopted. In addition to the choice of an appropriate set of keywords, it is thus important to (i) make these differences clear as we are interested in the vaccination schedule adopted by the author of the comment, and (ii) identify words which refer to the type of vaccination schedule (e.g., '*regular*'). Here we show the details of the pipeline which is constituted by a filter and a classifier.

2.1.1 Comment Filtering

The task of the filter is to retrieve sentences in which their authors wrote about the vaccination schedule they are following or they intend to follow. We need thus to focus on sentences containing words related to vaccination schedules and having as subject the author of the comment. The main tool we employ is the syntactic dependency parser provided by the open source library spaCy (https://spacy.io). Given a sentence, it produces a dependency tree that assigns parts-of-speech tags (e.g., noun, verb, adjective) to the words, and links them via syntactic dependencies. An example of the dependency tree of a sentence is shown in Figure 1. The main tasks of the filter are:

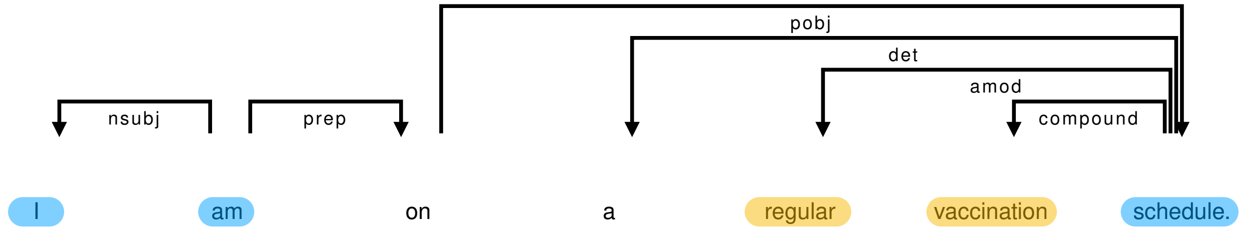

Figure 1: **Example of schedule-related sentence containing the word "*schedule*" and its syntactic dependency tree**. The arrows correspond to the dependencies between words whose labels are shown below them (for the full list of annotations of SpaCy https://spacy.io/api/annotation.)

1. **Keyword retrieval and dependency parsing.** We define contextual patterns to retrieve all the sentences containing at least one of the words pertaining to the schedule itself ("*schedule*") and its modality such as "*delay*", "*space*", and "*split*" along with their conjugations. Sentences containing a question mark ("*?*") are discarded. We then obtain the dependency tree of these sentences and we filter out sentences in which (i) the keyword "*schedule*" is the subject or the verb (e.g., "my schedule is busy", "I need to schedule an appointment with the doctor") and (ii) the other keywords do not occur as verbs. We refer to these retrieval patterns as "schedule_noun" and "delay_verbs" respectively.

2. **Structured summary.** Once the sentences containing the pattern of interest are identified, we proceed by extracting relevant words around the matched keywords: subject, verb, adjectives, possessives, compounds and negations. For the pattern "schedule_noun", we collect the verb, defined as the closest verb among the ascendants of the keyword "*schedule*" in the dependency tree, and its subject (respectively "am" and "I" in Figure 1). For the pattern "delay_verbs", we collect the subject and the direct object of the corresponding keyword. In addition, we collect adjectives, compounds and negations referring to these words. When the subject is missing, the clause is likely to be a subordinate clause and in this case we extract the subject and the verb of the principal clause. We also determine the tense of all the verbs. By identifying the words with these syntactic roles, we build a summary that consists of a structured summary of the sentence by means of its main elements.

3. **Blacklist filter.** As we saw in the second example in the beginning of this section, we are not interested in all the sentences containing such retrieval patterns. In order to filter out irrelevant matches, we manually inspected the words which occur most frequently in each syntactic role and we annotate whether these words occur in contexts relevant or irrelevant to vaccination scheduling. The inspection was done by reading a sample of matched sentences containing the specific word in the specific syntactic role. For example, "*friend*" is a word among the ones not to be considered as subject as we are interested just in the behavior of the author of the comment (e.g., "my friend is on a delayed schedule" does not convey information about the schedule behavior of the author of the comment). Another example of irrelevant words is "*nap*" occurring as a compound of the keyword "*schedule*". Based on these lists, we defined a set of rules through which the filter can identify matches that are

likely to be relevant to the vaccination schedule behavior of the author of the comment. In addition, we discard matches whose verb occur at past tenses as they may refer to past behaviors.

The output of the filter is a list of schedule-related sentences, along with their structured summary. Thus, the filter results in a high-precision selection of sentences related to the vaccination schedule behavior of the author due to the manual annotation of words occurring in relevant or irrelevant contexts. These sentences, along with the corresponding structured summary, are given as input to the classifier.

2.1.2 Comment Classification

For the pattern "schedule_noun", we manually inspected the most frequent adjectives and compounds associated to the keyword "*schedule*" and we labeled them depending on the different kind of schedule they refer (e.g., "*modified*" refers to alternative schedule, "*regular*" to the recommended one). The classifier assigns the label "*recommended*" by default and the label is changed if at least one adjective or compound refers to an alternative vaccination schedule. For the pattern "delay_verbs", all the matches are labeled as "*alternative*". As a final step, we check for negations which can change the overall meaning and for each negation we switch the label of the sentence. We then aggregate these labels in order to assign a unique label to comments, keeping only comments having matches labeled with the same label.

We now have a set of schedule-related comments labeled depending on the vaccination schedule followed by their authors. Now we need to aggregate them to propagate comments' labels to users. Before doing that, we ask if it is possible to identify users who changed their vaccination schedule behavior during their activity on the forum. This may be possible by searching for users who wrote more than one schedule-related comment and having different labels.

2.1.3 Behavior changes

There are 1666 users who wrote at least two schedule-related comments with different labels.

We represent each user as a sequence of binary values, where $+1$ and -1 refer to comments labeled as "*recommended*" and "*alternative*" respectively. The sequence lists all the schedule-related comments of an user in chronological order. Every switch in the sequence (i.e., from -1 to $+1$ or from $+1$ to -1) may correspond to a behavior change. For example, the sequence $[+1, +1, +1, -1, -1, -1, -1]$ can represent a behavior change because it is consistently $+1$ before the switch and consistently -1 after. Differently, the sequence $[+1, -1, +1, -1, -1, +1, -1]$ looks noisy and it is more likely due errors of the classifier. For this reason, we assume that behavior changes can be identified by searching for sequences displaying persistent changes. To quantify this property, we first define the *switch density* Σ as the number of changes of values in the sequence normalized by the length of the sequence minus 1. The *switch density* ranges from 0 to 1, indicating respectively uniform sequences and sequences in which $+1$ and -1 alternate. For example, the sequences shown above have respectively $\Sigma = 0.17$ and $\Sigma = 0.83$. To gauge whether this Σ could have happened by chance, for each sequence we shuffle the values one million times and estimate the probability of observing a value of Σ less than or equal to the one observed in the original sequence. We consider the change stable if this probability is less than 5% ($p = 0.05$). Only 10 out of 1666 users have a sequence satisfying this requirement and after manual inspection of their comments, we find only 5 who display a verifiable behavior change. Note that the length of these 10 sequences is no shorter than 8, meaning that we may miss behavior changes of less vocal users. We perform a spot check to see whether we can find shorter sequences with $\Sigma > 0$ that may signify a change of behavior. After manually examining 30 users with sequence lengths between 2 and 6, we find no users who describe actual behavior change.

This approach has some limitations, in particular due to the fact that short sequences are always discarded. In the following, we assume that users do not change their vaccination schedule behavior during their activity on the forum.

2.1.4 User Classification

Next, we use schedule-related comments as a proxy of users' behavior in order to classify their authors. However, due to the potential noise from the classifier we employ a null model to ensure the opinion of the users we study is stable and our inference is robust.

Among the 18 657 users who wrote at least one schedule-related comment, 29% (5400) wrote more than one comment. We assign a unique label to users defined as the most frequent label within their schedule-related comments. In order to discard noisy sequences, we compare the proportion of the least frequent label (within their comments) to the proportion that may be expected in a sequence of the same length due to the error rate of the classifier (as per manual assessment against the ground truth). Modeled with a binomial distribution, we assume that each classification is independent, and has a probability p of being erroneous. Users whose sequence has a higher proportion of least frequent labels than the one expected with 95% confidence level are discarded, resulting in a total of 1642 users removed. This method allows us to accept all uniform sequences independently of their length, while penalizing the shorter sequences among the non-uniform ones.

2.2 Experiences of AEFI extraction pipeline

We describe the pipeline developed to detect mentions of experiences of AEFI, and in particular we focus on first-hand or second-hand experiences of AEFI. In addition to retrieval patterns, previously used for the vaccination schedule extraction pipeline, we define other patterns called contextual patterns. These are needed because it is frequent to identify mentions to reactions which may be not related to vaccination. Let us consider these two examples:

"*Yesterday my son got his two months vaccine and during the day he had an high fever.*"
"*During the flu season, I always get fever!*".

In both cases, the author reports an experience of fever, one of the adverse event that can occur after a vaccine. However, in the first case it is a proper reaction because it is related to the vaccine ("*my son got his two months vaccine*") while in the second the fever is due to flu. Contextual patterns allow us to reduce the contribution of mentions to adverse reactions that are not attributed to the vaccine. In the following we show each step of the pipeline. As the structure is similar to the scheduling one, we just focus on the main differences.

2.2.1 Comment Filtering

Table 1 shows the dependency patterns (left column) and their accompanying keywords (right column) considered for the different dependency roles. These keywords were selected based on manual inspection of most frequent words occurring in the syntactic roles defined by the patterns. Thus, our extraction of mentions to experiences of AEFI takes into account frequently mentioned adverse reactions in our dataset. Note that contextual patterns are applied on the whole comment, if at least one retrieval pattern is matched. Figure 2 shows an example of a sentence triggering such patterns. When none of the contextual patterns are matched, we take into account the mentions of adverse events in the title of the comment's post to contextualize the comment. Then, we apply a blacklist filter and we discard all sentences containing question marks ("*?*") or one of the following words: "*if*", "*in case*" and "*unless*".

2.2.2 Comment Classification

By default, the classifier assigns the label "*reporting adverse events*" to all the retrieved sentences, and change the label to "*reporting no adverse events*" if a negation is matched. In order to assign an unique label to each comment (which may consist of several sentences), we aggregate the labels and code the comment as "*negative experience*" if it contains at least one sentence labeled as "*reporting adverse events*". Otherwise, the comment is labeled as "*positive experience*". In addition, we take advantage of the structured summary of

Table 1: **Retrieval and contextual patterns of the experience of adverse events following immunization pipeline**.

**Pattern**	**Words**
**Retrieval patterns**	
$\text{SUBJ} \xleftarrow{nsubj} \text{VERB} \xrightarrow{dobj} \text{REACTION}$	VERB in *cause, develop, do, experience, feel, get, give, have, notice, remember, report, run, see, show, spike, suffer*<br>REACTION in *arm, bump, change, damage, diarrhea, disorder, effect, fever, headache, injury, lump, nose, pain, rash, reaction, regression, seizure, spot, temp, temperature, vomit*
$\text{SUBJ} \xleftarrow{nsubj} \text{VERB} \xrightarrow{acomp/attr} \text{REACTION}$	VERB in *be, become, get*<br>REACTION in *crabby, cranchy, fussier, fussy, grumpy, irritable, lethargic, painfull**, *red**, *sleepy, sore, swollen, tired**, *warm*
$\text{SUBJ} \xleftarrow{nsubj} \text{react to} \xrightarrow{pobj} \text{SOMETHING}$	
**Contextual patterns**	
$\text{RET. PATT.} \xrightarrow{prep} \text{PREP} \xrightarrow{pobj} \text{VACCINE}$	RET. PATT. is any of the retrieval patterns defined above<br>VACCINE in *booster, dose, dtap, injection, mine, MMR, round, serie, shot, tdap, vac, vacc, vaccination, vaccine, vacs, vax*
$\text{PREP} \xrightarrow{pobj} \text{VACCINE}$	PREP in *after, from, since, whenever*<br>VACCINE in (as above)
$\text{SUBJ} \xleftarrow{nsubj} \text{VERB} \xrightarrow{dobj} \text{VACCINE}$	VERB in *get, give, have, receive*<br>VACCINE in (as above)

SUBJ : subject, PREP : preposition

The left column shows the patterns used to retrieve comments related to experiences of past vaccination in the filtering phase and the words on the right column are the keywords used for each syntactic role. Such keywords were chosen by inspecting all the most frequent words occurring as verbs or reaction. The ones most likely related to AEFI are chosen to build these lists. When the list of keywords is not shown, we collect all the words occurring in such role and use these words to develop the blacklist filter.
* These are general keywords which are retrieved only if the first contextual pattern is matched.

the matched sentences to extract the person who experienced the reaction and the specific kind of reaction. For the person, we label each kind of person as "*author*", "*author's child*" or "*acquaintance*", which allows us to differenciate between firs-hand experiences ("*author*", "*author's child*") and second-hand experiences ("*acquaintance*"). For the kind of reaction, we categorized the reactions we track in different cathegories (e.g., "*temp*", "*temperature*" belong to the cathegory "*fever*"). For example, the comment shown in Figure 2

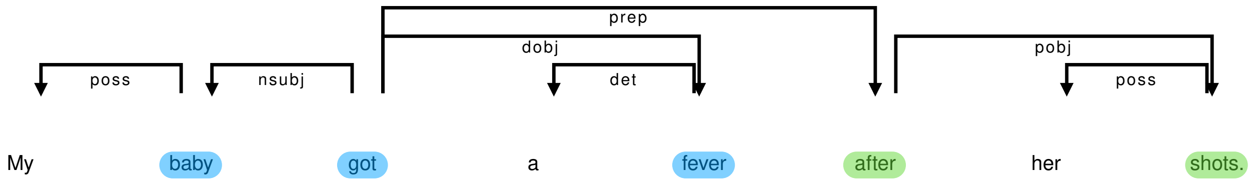

Figure 2: **Example of a sentence about adverse events following immunization**. This sentence triggers the first retrieval pattern and the first contextual pattern shown in Table 1 through the words highlighted in light blue and green respectively.

will be classified as ”*negative experience*”, the person who experienced the reaction is ”*author's child*”, and the adverse event experienced is ”*fever*”.

3 Geolocation

To assign a location to users, we use the self-reported location on the profile pages of users and the “local” groups joined by users (e.g., “Maryland Mommas”). We then aggregate these information to assign one of the 51 states and, when possible, a city to users. As self-reported locations and group names can contain acronyms, abbreviations, or colloquial names, we use a mixture of manual inspection and external resources for this task.

Geolocation from user profile pages. There are 66 708 users with self-reported location on their profile pages. We first inspect all the location text strings used by more than 10 users in order to find frequent abbreviations and manually map them to cities or states, ending up with 78 manually mapped strings (e.g., ‘philly’ is mapped to ‘Philadelphia, PA’ and ‘cali’ is mapped to ‘California’). To extract the locations for the remaining strings we use Nominatim (`https://nominatim.openstreetmap.org/`), a search engine for geo-referenced OpenStreetMap locations. By following this methodology, we are able to geolocate 64 792 users (29 375 of which at the level of city), which account for for 97% of the initial set of users.

Geolocation from local groups. There are 15 577 users who joined at least one of 19 289 local groups. Only 239 local groups are joined by more than 10 users, and cover 96% of the set of users. We thus proceed to manually map these groups to their corresponding location. For the remaining groups (joined by 527 users), we map them to states olny if their title contain the name of one US state. Overall, we obtain the mapping for 348 groups which cover 13 774 users. We first remove users who join groups mapped to different states (497 users). Then, the city is assigned to these users only if they do not follow groups mapped to different cities. Finally, we geolocate 13 306 users (5339 of which at the level of city), which account for 85% of the initial set of users.

Putting the information from profile pages and local groups together, we remove the users mapped to more than one state (749 users) and we keep the information related to the city only for users having unique city-level geolocation. This unification results in the final set of 65 423 users geolocated within U.S., of which 29 463 at city level. We do not consider cities as the result is very sparse, with only 50 cities with more than 100 users.

To evaluate the geographic representativeness across the states, we compute the Pearson correlation coefficient r between the log of the number of users assigned to each state and the log of the 2010 US census population [`https://www.census.gov/data/tables/time-series/demo/popest/2010s-state-total.html#par_textimage`]. This results in $r = 0.96$ ($p \ll 0.01$), thus showing a good representativeness of BabyCenter users.

4 Construction of the interaction network

To represent the interactions of users, we construct a network where each node represents a user and there is a link from user i to j if i comments on a post by j (considering all vaccine-related posts and comments, not only those about scheduling). The result is a directed network where the links are weighted by the number of comments one user has made on another user's posts. We begin with a network with 201 208 nodes and 642 992 edges. We then remove self-loops and nodes with degree less than 5, to limit the contribution of noise, and consider the giant connected component (encompassing 99% of the nodes), resulting in the final network with 55 900 nodes and 409 172 edges.
