# Supplementary figures and images for: Detecting adherence to the recommended childhood vaccination schedule from user-generated content in a US parenting forum
Source: PLoS Comput Biol. 2021 Apr 26;17(4):e1008919. doi: 10.1371/journal.pcbi.1008919 (PMC8075195; doi:10.1371/journal.pcbi.1008919)

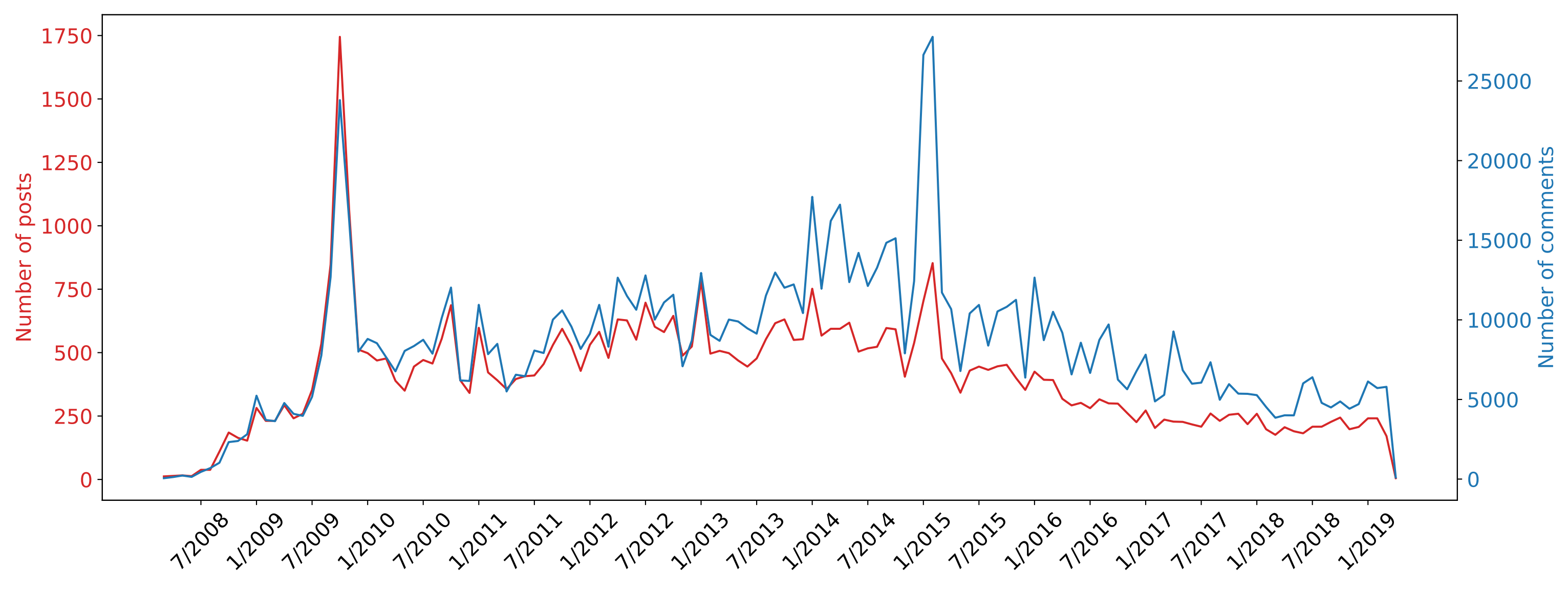

Supplement: S1 Fig — Number of posts (red) and number of comments (blue). (PDF) [file pcbi.1008919.s002.pdf]

Distribution of number of posts per user

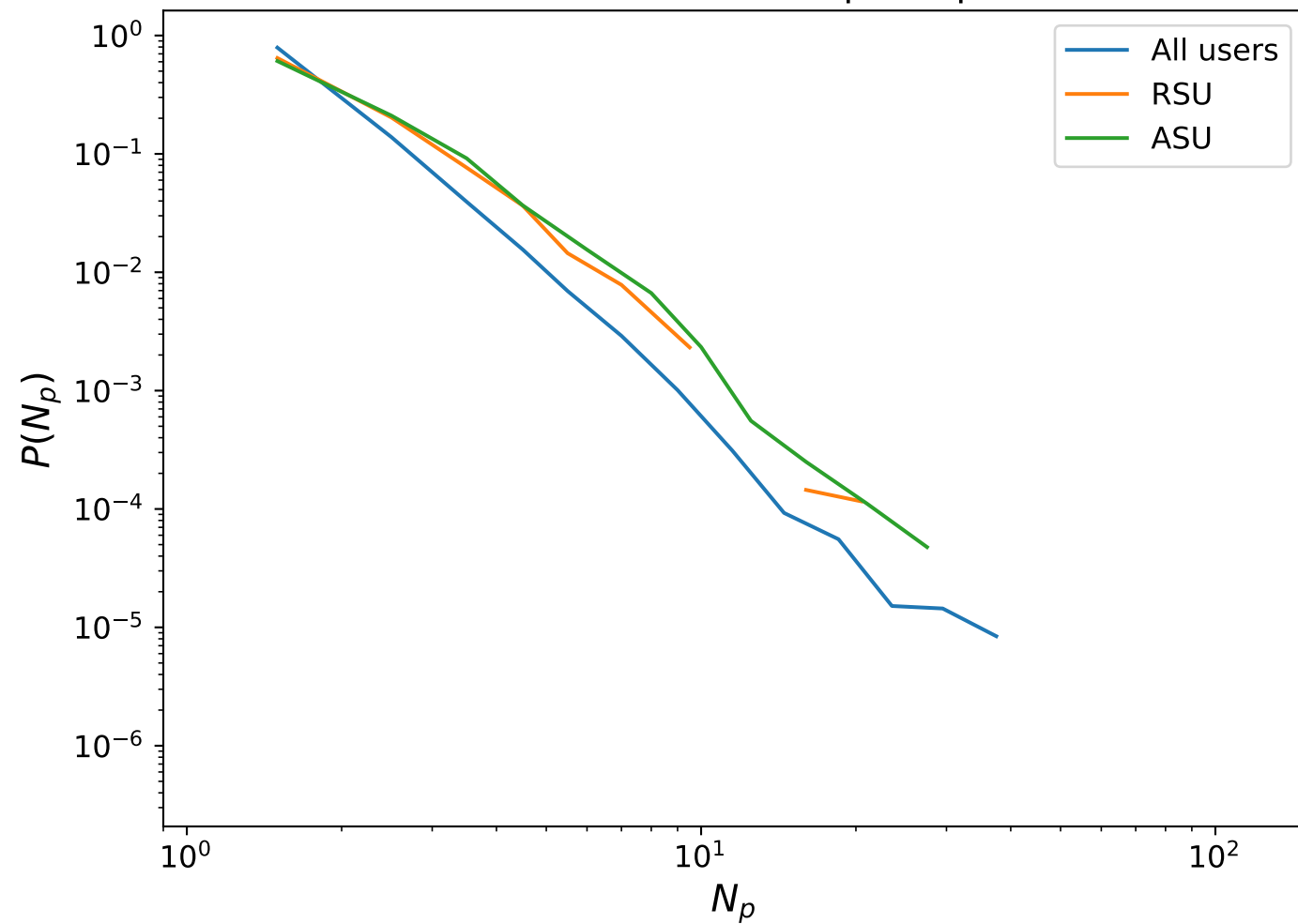

Distribution of number of comments per user

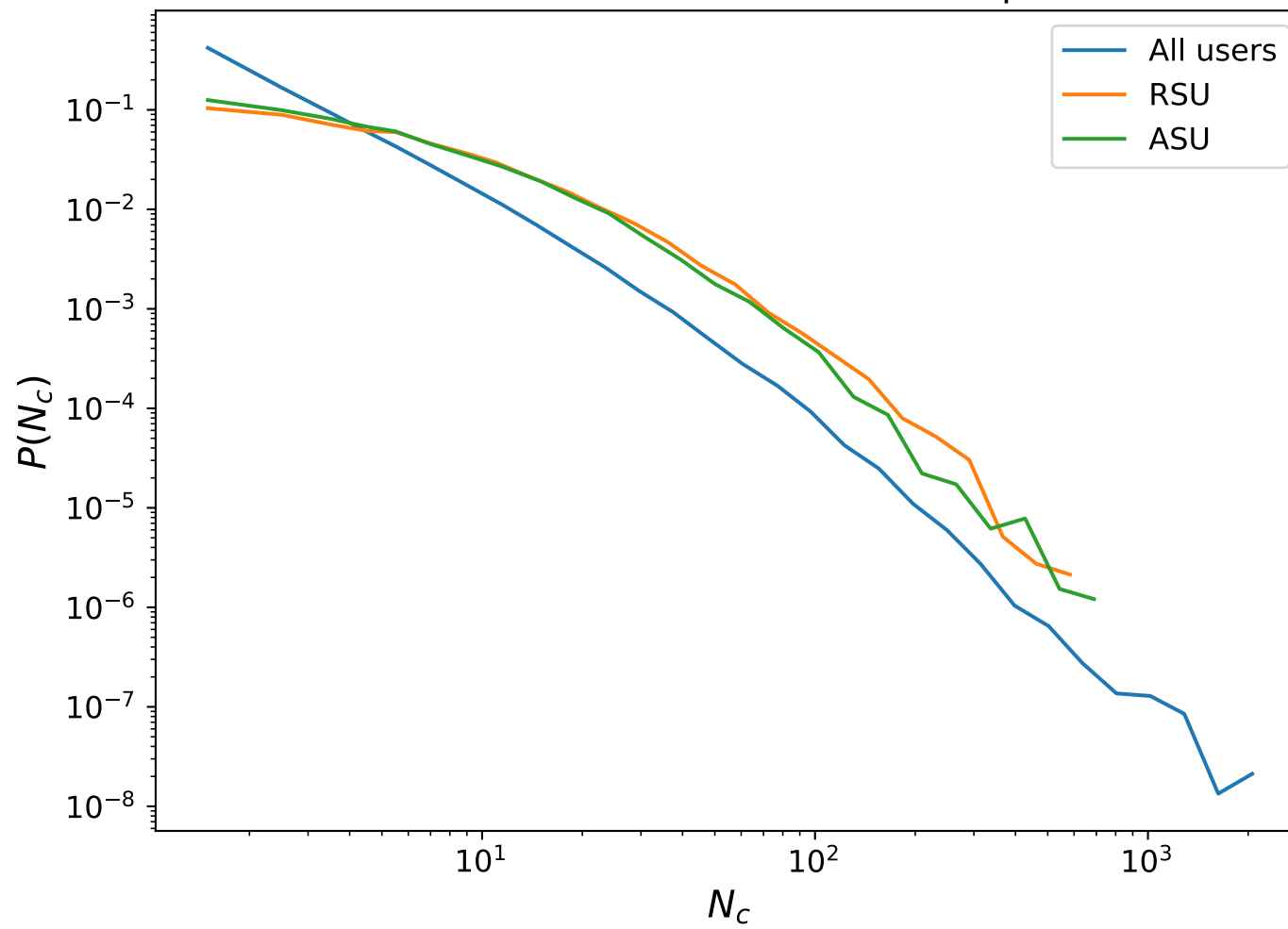

Supplement: S2 Fig — The distributions for all users, regular schedule users (RSUs) and alternative schedule users (ASUs) are shown. (PDF) [file pcbi.1008919.s003.pdf]

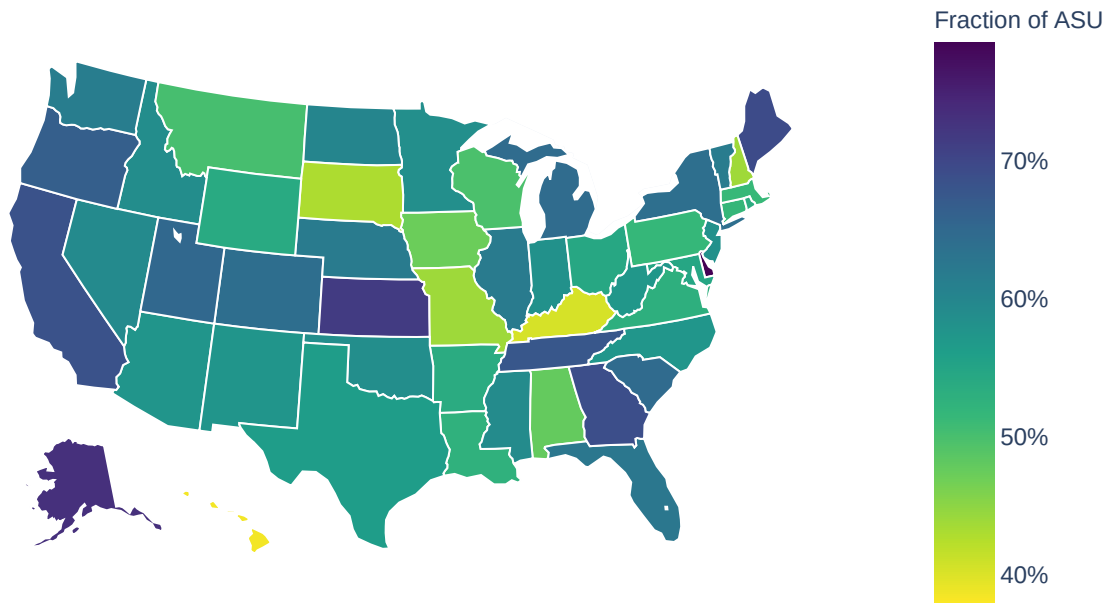

Supplement: S3 Fig — The authors created the map based on the built-in geometry of the open source Python library Plotly (https://plotly.com/python/). (PDF) [file pcbi.1008919.s004.pdf]

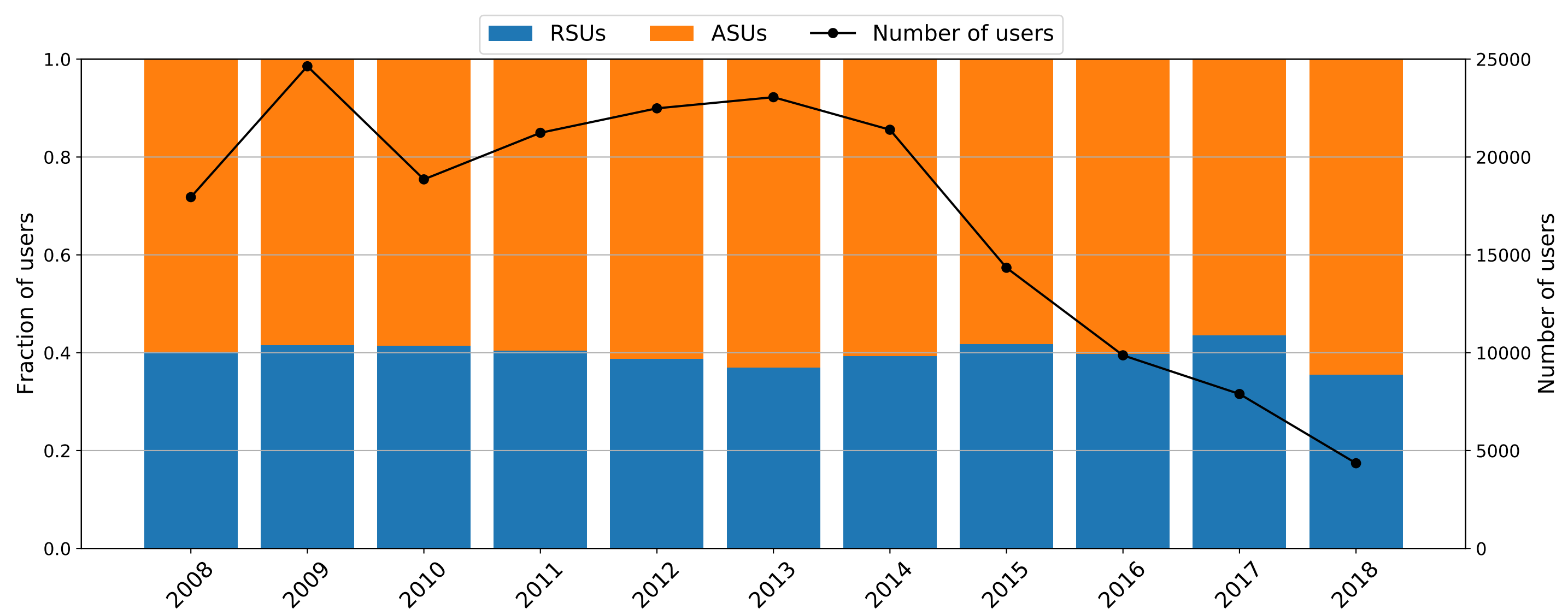

Supplement: S4 Fig — The solid line indicates the number of users who joined the forum yearly, while vertical bars show the distribution of regular schedule users (RSUs) and alternative schedule users (ASUs) among new users. (PDF) [file pcbi.1008919.s005.pdf]

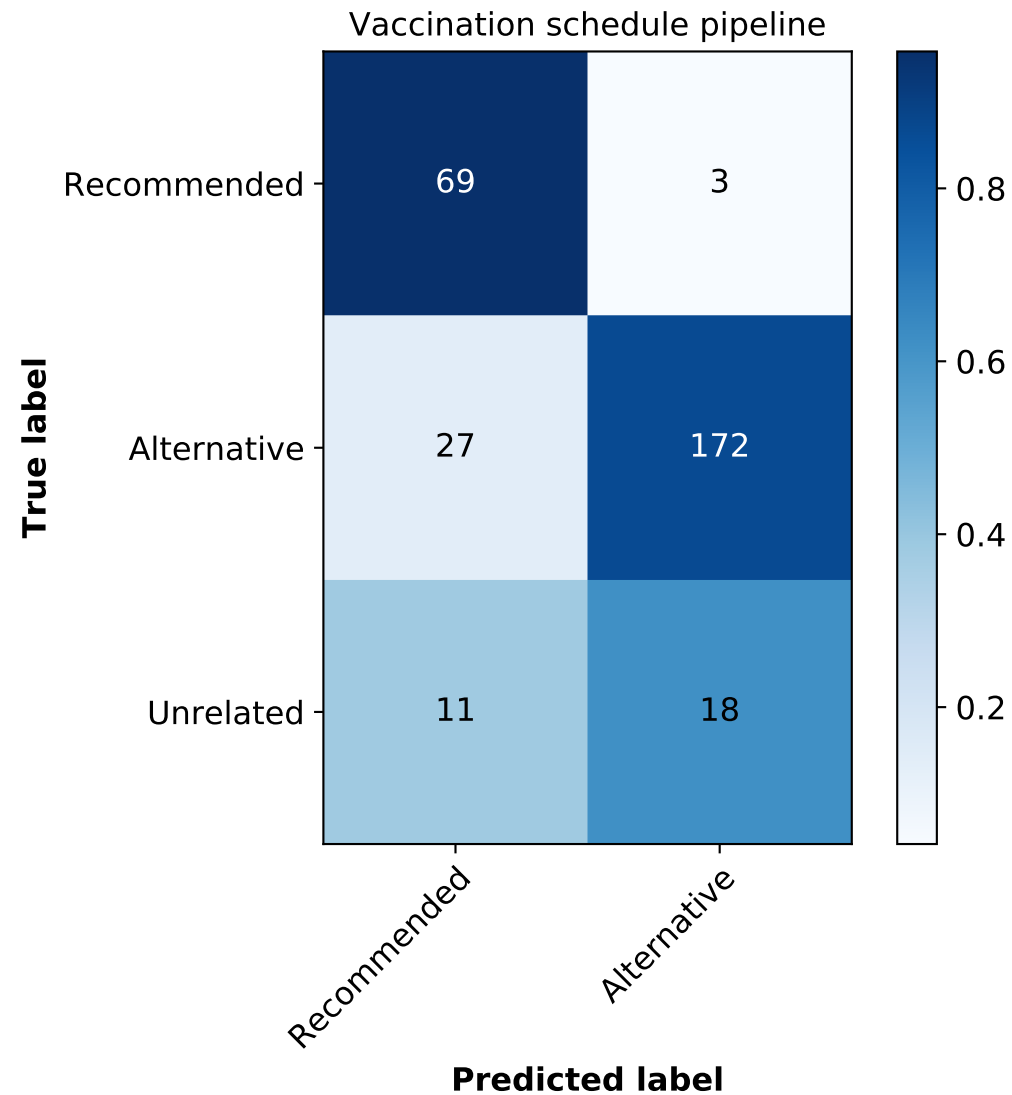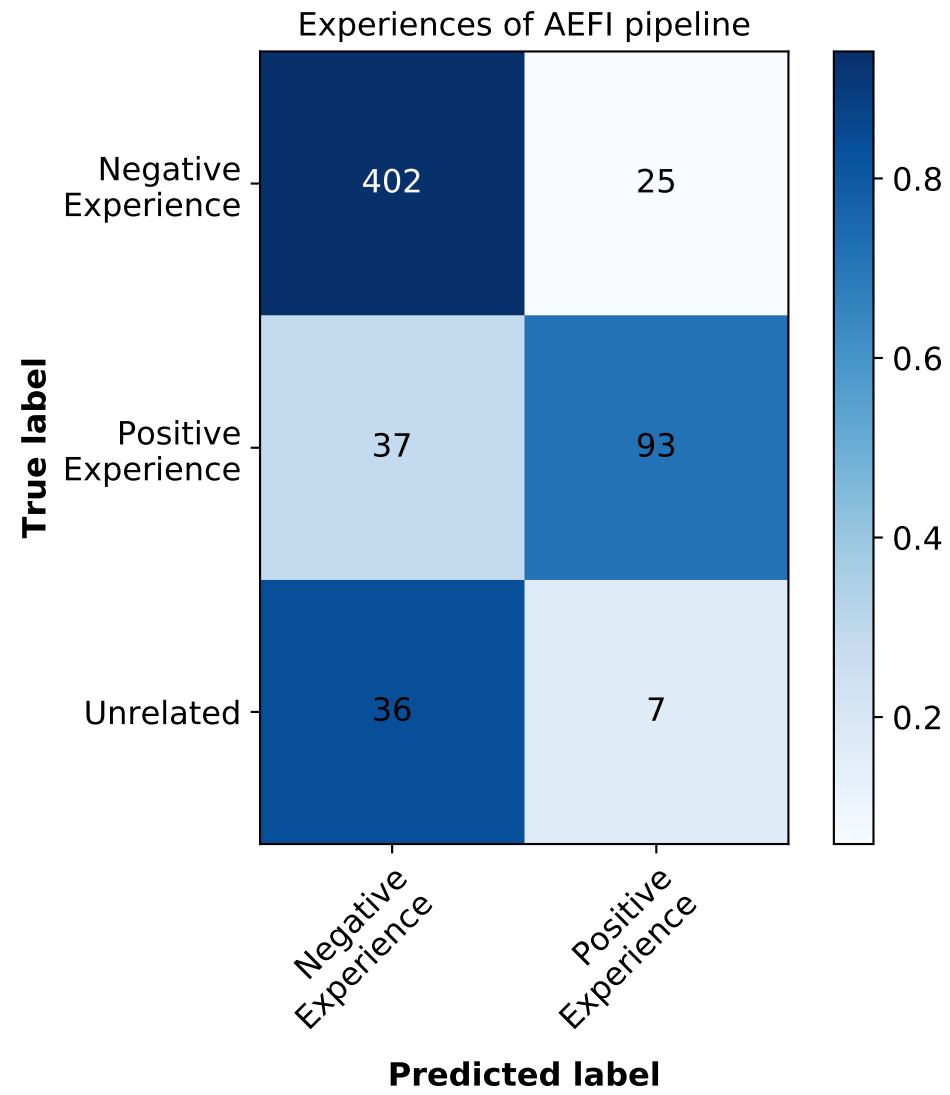

Supplement: S5 Fig — Raw numbers are shown within each cell. (PDF) [file pcbi.1008919.s006.pdf]
