# Supplementary material for: Detecting adherence to the recommended childhood vaccination schedule from user-generated content in a US parenting forum
Source: PLoS Comput Biol. 2021 Apr 26;17(4):e1008919. doi: 10.1371/journal.pcbi.1008919 (PMC8075195; doi:10.1371/journal.pcbi.1008919)
Supplement: Attachment — Submitted filename: Response_to_reviewers.pdf [file pcbi.1008919.s008.pdf]

1. Replies to the comments of the First Referee

Thank you for the opportunity to review this manuscript, which seeks to characterise and compare two types of users. The strategy for selecting users from two groups is a reasonable one (but isn't as reflected in the results as it could be), the methods are generally transparent and replicable, but there are some minor issues with the framing and the attempt to draw conclusions about the implications of the work.

We thank the Referee for carefully reading our manuscript and for the constructive comments provided, which we address in the new version of the manuscript and discuss in detail in the following paragraphs.

Overall: As with all other studies of individual online forums, the participants are self-selected and represent a special subgroup of people. Some of those people are over-represented as users in online forums. As a consequence any data analysis is fundamentally limited by an obscured view of a very biased sample of the population and this limits the conclusions that can be drawn. Suggesting that data from a forum can be used to support the development of digital interventions is problematic because there is an implicit assumption that (a) the results will generalise over time and beyond the forum, and (b) that people who openly discuss or advocate for ASUs are the right "target" for digital interventions (people reading without participating are more likely to benefit from interventions - more below).

We acknowledge that this sample of users has several biases and we do not aim to generalize our conclusions over time, nor beyond the forum. In this work, we assessed the vaccination behavior of a subset of users (the ones stating their vaccination behavior) and we studied their activity on the forum, showing interesting findings. Even though we did not argue that our findings can be generalized, we argue that being able to extract such information can be an additional instrument for policy makers who want to develop intervention campaigns on the specific platform under analysis. Different platforms have their own normative biases and we believe that a deeper understanding of the users participating on a specific platform (and thus the same ones who may benefit from digital intervention) can better inform intervention strategies and reduce the chances of backfire effects.

Regarding the right "target" for digital intervention, we can not exclude that people reading without participating may benefit from digital intervention, even more than users who follow alternative vaccination schedules. Unfortunately, we have not this kind of data, and every conclusion about these users would be only speculative. The data we were able to collect allowed us to focus only on users sharing their vaccination behavior with the community.

In light of this clarification, we have rephrased the last sentence of the Abstract and added a sentence regarding people reading without participating in the Discussion section:
"While our findings are limited to the specific platform analyzed, our approach may provide additional insights for the development of campaigns targeting parents on digital platforms." (Revised manuscript: Abstract)
"In addition, we have no information about users who read the content published on the forum without participating, another group of individuals who may benefit from intervention campaigns." (Revised manuscript: lines 325:327)

Abstract: Nice to see an abstract that includes details about the dataset, what was done to it, and clear description of findings. The only issue was the sentence starting with "Though", which does not read well. As above, I also disagree with the implications sentence and I think it can be dangerous to infer from a special subset of people who post on forums about what the population thinks and feels.

We thank the Referee for their comments about the clarity of the Abstract. We rephrased the sentence starting with "Though":
"Content analysis of comments shows that the resources most frequently shared by both groups point to governmental domains (.gov)." (Revised manuscript: Abstract)

We have also modified the last sentence of the Abstract as described in the previous answer.

Author Summary: I do agree with the last part of this section and I think this is a more nuanced statement around the capacity to enhance communication between people who promote (or simply make) different

decisions. Is there any risk that this will backfire and increasing communication will instead increase hesitancy and refusal where certain groups become more persuasive for the "audience" (not the people posting but the people reading the posts)?

We agree with the referee that interventions may backfire. We are not aware of works that study how the "audience" respond to intervention campaigns on digital platforms. However, an evaluation of the possible intervention strategies, along with their drawbacks, that can be used on BabyCenter US is beyond the scope of the present work.

We also want to clarify that intervention campaigns can be structured around different strategies than the enhancement of communication between people who promote different decisions. As an example, BabyCenter has an editorial team that create contents with the supervision of an advisory board of doctors [1], addressing subjects like infant health. These contents might be created based on the concerns of a group of users or recommended to users who follow alternative vaccination schedules. Another example is *r/IAmA*, one of the most popular Reddit's communities where experts are interviewed and users can ask questions to them through comments.

Introduction: It wasn't clear why the focus was only on the US when this is a global problem and I assume the forum is not restricted to US posters and US audiences?

Introduction: Very nice to see that the work is guided by a conceptual framework rather than something simple or something reinvented.

We thank the referee for his/her comment about the conceptual framework we used to guide the work.

We acknowledge that vaccine hesitancy is a global problem. The forum is available in nine different languages (e.g., Spanish and Portuguese) and for other English-speaking countries (e.g., Canada and UK). In this work, we focused on BabyCenter US, but we could have taken advantage of the other forums used by parents from other countries. In the following, we will explain (i) why we considered only BabyCenter US, and (ii) why we argue that, despite BabyCenter US is not restricted to US posters, the activity of the forum is mainly driven by parents from the US.

The determinants of vaccine hesitancy varies across places and we opted to restrict the focus to the US in order to limit the cultural context under analysis. This allowed us to avoid additional confounding factors. Another more technical aspect to be taken into account regards the development of the extraction pipeline. This methodology strongly relies on language, syntactic patterns, and keywords that are frequently used by users in their comments. Restricting the focus made possible to develop the first version of the extraction pipeline, that can be properly modified to deal with other linguistic characteristics of other English-speaking countries and other languages. We plan to work in this direction with other studies.

Even though BabyCenter US is not restricted to users only from the US, we can reasonably assume that the vast majority of users are from the US. We are confident with this assumption because of the following reasons: (i) almost all the geolocated users are located in one of the US states; (ii) by looking at the activity on the forum (S1 Fig) we can see two peaks of activity that can be related to two events happened in the US (start of the H1N1 vaccination campaign in the late 2009 and the spreading of measles from the Disney theme park in California in early 2015), pointing to the fact that the bulk of the activity on BabyCenter US is driven by parents from the US; (iii) users from different countries have other spaces to join where they can find other users from the same country (e.g., Brazil, Germany, India).

We have addressed the aspect of the audience in the previous answers and we remark that, despite it would be very interesting to study the behavior of the audience, we have no information about them and every consideration would be purely speculative.

We have substituted all the mentions to "BabyCenter" with "BabyCenter US" and we have added in the Data Collection section that BabyCenter.com is available in different languages and that we focused on BabyCenter US:
"BabyCenter.com is a popular parenting website available in nine different languages whose contents reach 100 million people monthly [2]."(Revised manuscript: lines 56:57)

"In this study, we focused on BabyCenter US."(Revised manuscript: line 62:63)

Data Collection: Typo "Babycenyer"

Data Collection: I read the statement that the data could not be released to the public but the method of data collection seems to suggest the website was crawled. This would be the appropriate place to note that this was aligned with the terms of service for the website, or that permission was granted, and evidence that human ethics was granted or waived by the university. I would not want to be PLOS Computational Biology if a user reads the paper and decides they see themselves in the data if it hasn't at least been checked by an ethics committee.

We thank the Referee for the suggestion to mention in this section that our data collection is in compliance with the Terms of Use of the forum. We have also remarked that the data collection consists in passive information gathering, in which we did not made any kind of intervention on the forum or interactions with users. Moreover, we collected only information that are publicly available and for which the identity of users can not be ascertained. Therefore, our research did not meet the regulatory definition of research with human subject, according to The Common Rule (45 CFR 46).

For example, similar work published in PLOS ONE and cited in the manuscript is based on data collected from a parenting forum similar to BabyCenter US [3]. The authors state that *This study was deemed exempt from review by the University of Pennsylvania Institutional Review Board due to the fact that it did not meet the regulatory definition of research with human subjects, as the data was obtained from publicly available sources.*

We have added the following statement in the end of the Data Collection section:
"We verified that our data collection was in compliance with the Terms of Use of BabyCenter.com [4] and we contacted the BabyCenter Community Team to inform them about our research. The Community Team forbids to interact with users and share contents on the forum. We remark that intervention and interaction activities are outside the scope of this work."(Revised manuscript: lines 68:72)

Determining adherence: Nice to see that multiple independent annotators were used and they were blinded to the output of the classifier label to check for correctness.

We thank the Referee for his/her comment about the evaluation strategy used in our work.

Activity of users: I think it is potentially dangerous to look at geographic representativeness. While it isn't a bad thing on its own, it starts to make it look like the work is headed towards a "representative sample" of the United States. There are so many reasons why people who decide to post on a forum are never going to be representative. Looking at location might give readers a false sense of representativeness that absolutely does not exist. It would be much better to look at the representativeness of the "audience" and not the users posting.

(As evidence, 201K users posted comments over 11 years but the website supposedly reaches 100 million people; which means you get a very biased 0.2% of the audience. It makes it much worse that ASUs are heavily over-represented on the forum when this is not at all reflected in the population. Clearly people promoting vaccine choice/hesitancy/etc. are much more vocal.)

We agree with the Referee on this point. Indeed, it is important not to give the reader the impression that this sample is a representative sample of the US population. To do that, in the Activity of users section we have mentioned "geographic distribution" instead of "geographic representativeness". We have modified accordingly also the Results section and we have added a sentence to make clear to the reader that, despite the high correlation, we can not establish if our sample of users is representative of the US population, especially on characteristics beside geography.

In Results section now:
"We found the geographical distribution of users mentioning vaccination scheduling (RSUs + ASUs) to be highly correlated with the distribution of the US population across states ($r = 0.94$, $p < 0.0001$). Nevertheless, we have no additional information to asses if this group of users is a representative sample

of the US population."(Revised manuscript: lines 210:214)

Results: the first does not read well "96% of which with at least one comment,"

We have changed this sentence:
"We downloaded 54,227 posts related to vaccines and 1,129,487 comments from a total of 201,986 unique users. Only 4% of posts have no comments." (Revised manuscript: lines 178:179)

Results: The way the study is set up is that two groups of users were to be compared in terms of their behaviour on the site, but then the results are mostly about characterising the whole group.

Our work is aimed at characterizing both the behavior of the whole group of users and to highlight the differences between the behaviors of the two groups identified.

Results: The groups analysis and Figure 2 are very nice and likely "actionable" in the sense of understanding where audience/users are at higher or lower risk.

We thank the Referee for his/her comment about the clarity of Figure 2. We have also augmented the figure with color-coded themes, as suggested by another referee.

Discussion: :"Out of these, 60% expressed an intention to follow an alternative vaccination schedule (ASUs) a much higher fraction than the one estimated by surveys [26]. This may be due to ASUs being more vocal about their scheduling choices (see Figure 1)." This is simply the wrong comparison, or perhaps just the wrong wording. Obviously the proportion of users commenting about schedules on a forum bears no resemblance to the underlying distribution or clustering of hesitancy in the population and the comparison doesn't make sense - instead it might be worth including both numbers in the same paragraph to say clearly that people promoting ASUs are vocal on this particular forum and this is obvious because it is completely unaligned with prevalence.

We thank the referee for this comment, which we agree with. For this reason, we have rephrased the sentence as suggested, avoiding direct comparison:
"Out of these, 60% expressed an intention to follow an alternative vaccination schedule (ASUs). This group is more vocal than RSUs when writing about vaccination schedule (see Figure 1) and this may account for a possible underestimation of the number of RSUs. On the other hand, this may be a potential benefit for intervention campaigns as it is easier to identify users following an alternative vaccination schedule." (Revised manuscript: lines 285:290)

Discussion: The finding that there is frequent communication between the groups is interesting and worth focusing on.

Reply: We're glad the referee has found the insight interesting.

Discussion: The note about representativeness buried in the discussion is not enough in my opinion. Readers will ignore this limitation when citing or describing this work.

As stated in the previous comments, we agree on the importance of making this aspect clear. We have added a sentence in the Results section stating that we have no information to assess the representativeness of users with respect to the US population.

2. Replies to the comments of the Second Referee

In this work the authors analyse posts extracted from a public online forum (babycenter.com), on which current or prospective parents exchange information about childcare. The authors used a Natural Language Processing approach to detect posts related to vaccinations and determined whether these posts were by parents following a recommended (RSU) or alternative (ASU) vaccination schedule for their children. The authors also determine whether the posts are about specific adverse effects of vaccinations. They then compare RSU and ASU groups for differences in the type of content they share and find that ASUs are more likely to focus on alternative medicine and mention more severe adverse effects. They also explore the presence of echo chambers on the site and find that the two groups communicate with each other, however ASUs express stronger homophily. The work ends with specific recommendations for targeted public health interventions on online media, such as forums.

I find the work interesting as it gives insight on the concerns regarding vaccinations by parents. By analysing a relatively large dataset in an automated way, the authors are able to confirm several findings from surveys. As the work shows, online forums can therefore be an interesting public data source for answering specific questions regarding attitudes towards vaccination. My comments are mostly minor:

We thank the Referee for carefully reading our manuscript and for finding our work interesting. The Referee also provides several constructive comments, which we address in the new version of the manuscript and discuss in detail in the following paragraphs.

The authors state that 7 annotators have manually labelled 300 random comments and proceed to implement a fairly complex rule-based classifier. The authors don't mention a split into a validation and leave-out test set. If the authors used the same set for validation and testing then I would expect the performance scores to be overestimated, as complex rule-based algorithms have a tendency to overfit

We did not used the manually annotated comments to define the rules of the extraction pipeline, but we employed them only for the final evaluation of the performance of the pipeline. In other words, the annotated comments were not seen during the developmental phase. We also agree that the performance of our pipeline may hardly generalize both on other domains and with newer data from the same forum, and this is not a purpose we aimed to reach.

The label distribution for the manually annotated data is not given. Is there label imbalance?

We have added the confusion matrices as supplementary figure (S5 Fig). In both cases, the label imbalance of the manually annotated data resembles the imbalance of the automatically classified data.

Similarly, performance scores should be provided for all estimated classes.

Besides precision, the metric used to evaluate the performance of the filter, we provided true positive rate (sensitivity) and true negative rate (specificity) to evaluate the classifier, where *alternative* is considered as the positive class. These two metrics describe how well the pipeline can identify true positives (i.e., *alternative* comments) and true negatives (i.e., *recommended* comments). Analogously, we considered the label *negative experience* of the experiences of AEFI pipeline as the positive class.

We have added two sentences in the Materials and Methods section specifying which label we considered as positive class to compute sensitivity and specificity of both the pipelines:
"We employed Cohen's Kappa κ to measure inter-annotator agreement. The performances of the filter and the classifier were evaluated separately: first we computed the precision of the filter in the retrieval of relevant comments, then we computed the sensitivity and the specificity of the classifier after discarding unrelated comments (considering the label alternative *as the positive class)."* (Revised manuscript: lines 100:104)
"We evaluated this pipeline in the same way as discussed in the previous section (considering the label negative experience *as the positive class), with the exception that in this case we manually labeled 600 comments."* (Revised manuscript: lines 148:150)

"RSUs posted more schedule-related comments per user compared to ASUs" (line 173). This seems to

contradict the data in table 1 and the discussion (line 258).

The two acronyms were inverted. We have change the text by replacing "RSU" with "ASU" and vice versa.

I'm missing some information regarding content moderation. Are extremist views removed from the site? If so, under which circumstances.

The forum is a place where parents can freely share their opinions and experiences. Differences in philosophies, beliefs, opinions and parenting styles, including "extreme" opinions about vaccination, are welcome on the forum as they enrich the discussion with different point of views. The staff of BabyCenter reserves the right to ban users or remove contents under specific circumstances including personal attacks and hate speech.

We refer to the following resource for additional information regarding the community guidelines:

Community guidelines: `https://www.babycenter.com/community-help-guidelines`

We have added the following sentence in the Data collection section:
"Users are free to express their opinions and moderators can remove contents under specific circumstances that include hate speech, personal attacks and illegal activities [4]." (Revised manuscript: lines 59:61)

I have a hard time understanding the long sentence line 308-312. Maybe should be split into multiple sentences.

We have split the sentence:
"These limitations, however, complement those of traditional survey-based methods. For example, it is possible to capture ongoing discussions which happen among fellow parents, potentially reducing recall, motivated forgetting and conformity biases. Moreover, topics of discussion are not limited by a surveyors predetermined set of hypotheses." (Revised manuscript: lines 339:343)

The term "hashtag-able" might not be understood by all readers (line 319).

We have paraphrased this term:
"(instead of simple messages based on hashtags that are the current norm)" (Revised manuscript: lines 349:350)

"Differently from what is observed on most popular digital platforms like Facebook or Twitter, where users communicate only with like-minded user" (author summary). "Only" is too strong here.

We have substituted "only" with "mainly". (Revised manuscript: Author summary)

"We downloaded 54,227 posts related to vaccines, 96 of which with at least one comment, and 1,129,487 comments from a total of 201,986 unique users." (line 162/163) - a bit hard to read (again, should be split into two sentences).

We have paraphrased this sentence:
"We downloaded 54,227 posts related to vaccines and 1,129,487 comments from a total of 201,986 unique users. Only 4% of posts have no comments." (Revised manuscript: lines 178:179)

The dataset spans more than 11 years. Could a temporal trend be observed? Could this data source be used for monitoring?

Despite the long time span of the dataset, we can observe no temporal trends due to the low number of comments per month from RSUs and ASUs. The activity of RSUs and ASUs in terms of comments and posts resemble the activity of all users on the forum (S1 Fig). Even though it might be of interest to monitor how users react to exogenous events (e.g., the change on vaccination exemption policies in California and Vermont), the volume of data is too low to extract meaningful conclusions. The same conclusion holds if

we want to study the temporal evolution of mentions of adverse reactions following immunization (both aggregate and at the level of states).

Regarding the temporal trend of the activity of all users (not only RSUs and ASUs), we can see two peaks of activity (S1 Fig). The first can be associated to the launch of the national influenza H1N1 vaccination campaign (October 2009) and the second to the measles outbreak linked to the Disney theme park in California (from January 2015). Other than that, the trend of the data across time shows an increase in activity from 2008 to 2011 and a decrease of activity after 2015, which is likely related to the popularity of the forum [3]. Note that our dataset consists of posts and comments related to vaccination, making it difficult to disentangle the evolution of the popularity of the forum from the varying interest of users toward vaccination.

In our opinion, this data source may be useful for monitoring sentiment about vaccines. For instance, studying how users react to exogenous events related to vaccination (e.g., changes in policies, introduction of new vaccines). Due to the possible kind of biases this source of data may be affected, all the conclusion that follow from the monitoring hardly generalize outside this specific platform.

We have added a description of the trend of the activity of all users in the Results section:
"S1 Fig shows the monthly volume of posts and comments in which two peaks of activity are visible: the first in late 2009 and the second in early 2015, probably related to the the start of the H1N1 vaccination campaign and the spreading of measles linked to the Disney theme park in California respectively." (Revised manuscript: lines 180:184)

The introduction doesn't cover whether studies exist in which similar forums have been analysed. I would also count Reddit or Gab as more discussion-based forums in this context.

We have added two sentences in the Introduction section, mentioning both other studies on similar forums and on BabyCenter:
"Several studies used content analysis to determine topics of discussion, narratives and vaccine-related discussion topics on digital platforms like Reddit or parenting fora [5, 6, 7]" (Revised manuscript: lines 31:33)
"Other studies used this source of data to investigate themes related to vaccination [8] and other topics[9], but none of them performed an analysis at the level of users." (Revised manuscript: lines 43:45)

Although it is understandable that the raw data cannot be made public, I was wondering whether certain aggregated datasets could be provided. In particular the interaction network between (Fig. 5) could be of interest.

We have checked the Terms of Service of BabyCenter.com and we will share the whole interaction network. For further information on the location of this dataset we refer to the Data Availability statement.

The choice of the singular search term "vaccine" (and not a more complex regex pattern) is a bit unfortunate - however the authors have already partially hinted at this issue in the discussion (line 297).

We acknowledge that a single search term may result reductive and the consequent coverage of retrieved posts may be only partial. However, we have no information on how the search function of the forum works and how it would manage more complex search queries.

A posteriori, we checked for the results obtained from this search and we observed that the retrieved posts contain also words like "vaccines" and "vaccination". This leads us to think that all the posts containing the stem of the search term ("vaccin") may be retrieved.

We have added the following sentence in the data collection section:
"In addition to the search term 'vaccine'*, the retrieved posts contain also words whose stem is* 'vaccin' *(e.g., vaccines, vaccination)."* (Revised manuscript: lines 184:185)

I've had difficulties following the term "class length" (line 178). If I understood correctly this is simply the number of schedule-related comments. Maybe something more descriptive than "class length" could

be used in the text. Also, Figure 1 is missing the corresponding x-axis label.

We have changed the term "class length" in the Materials and Methods, and Results sections and we have added the label of the x-axis of Figure 1:
"To do this, we divided schedule-related comments in classes $C_{(a,b]}$, so that schedule-related comments written by users who wrote a number of schedule-related comments greater than a and lower or equal than b belong to the class $C_{(a,b]}$." (Revised manuscript: lines 114:118)
"Figure 1 shows that the estimated probabilities to find a schedule-related comment coded as recommended *decreases for more prolific users."* (Revised manuscript: lines 203:205)

What does the horizontal line in Fig. 1 mean? Is it simply the mean over all data?

Yes, it is the fraction of schedule-related comments labeled as '*recommended*'. We have added a sentence in the description of Fig.1:
"The horizontal line corresponds to the fraction of schedule-related comments labeled as recommended*."*
(Revised manuscript: Figure 1)

3. Replies to the comments of the Third Referee

The authors present an analysis of data collected on a popular parenting forum, to detect adherence to recommended vaccination schedules among users and identify factors associated with it. Below are my comments:

We thank the Referee for carefully reading our manuscript and for providing several constructive comments, which we address in the new version of the manuscript and discuss in detail in the following paragraphs.

l78: "comments are labeled as ‘recommended‘by default". Can the method be applied with no "default" label, and check how many patients are then labeled in the ASU and RSU groups? Or in other terms, what is the proportion of users that clearly state which schedule they follow, and can we reasonnably assume that the ones not clearly stating it belong to the RSU group?

The answer to the first question is no, the method (in particular the classifier) can not be applied with no "default" label. At this specific point of the pipeline, we assume that all the filtered comments clearly state the vaccination schedule followed by the author (this is confirmed by the high precision of the filter). Then, we assume that all these comments can be classified as '*recommended*' by default, except when specific keywords are identified.

Also the answer to the second question is no, we can not reasonably assume that users not clearly stating their adherence to a vaccination schedule belong to the RSU group. If we wanted to make this assumption, we would have no information to asses if it is reasonable because these users did not mentioned the vaccination schedule they follow. Thus we choose to work only with the assertions that can be verified directly via the available data.

l165: "54% of users whose profiles were still active and public". It is not completely clear to me in which part of the analysis the full data of all users was used and in which part it was only reduced to the 54% subset. Is the distribution of ASU and RSU in this subset similar to the one in all of the users?

We acknowledge that this may have created some confusion to the reader and we have modified some sentences to make clear which subset of users was used for the corresponding analysis. We used five features to describe the activity of RSUs and ASUs on the platform, two of which can be determined from the user profiles pages, namely geolocation and groups. The other features were determined from users' comments. However, all the results were obtained considering the subset of RSUs and ASUs for which the feature under analysis is available (we provided the information about the number of users for each of these subsets in Table 2). The distribution of ASUs for each of these subset of users ranges from 58.59% and 63.29%, compared to 59.54% which is the overall fraction of ASUs.

We have added a clarification in the Materials and Methods, and Results section:
"Note that geolocation and the groups followed by users can be determined from user profile pages. This limits the corresponding analysis to the set of users whose profile page is publicly accessible. The other features were extracted from users' comments." (Revised manuscript: lines 127:130)
"The proportion of ASUs ranges from 58.59% and 63.29% for each of the features." (Revised manuscript: lines 207:208)

description of the ASU and RSU data: I think some additional points could be added to the descritive analysis to the data. In particular, if the geolocation of some users is available, could we have an idea of how ASU vs RSU are localized in the US? In addition to Fig 1, could we see the distribution of the number of comments per user, among all users and then ASU vs RSU? It would give some insights in term of behavior of users.

In addition, do you have access to other user-specific variables, such as gender or age?

For users, can you obtain the date at which they joined the forum, and if yes, is it possible to see the distribution of date at which users joined, among all users and ASU vs RSU? Did more ASU join in the more recent years?

We have added three figures as supplementary material: S2 Fig, showing the distributions of the number of posts and comments per users (all users, RSU and ASU), and S3 Fig, showing how the fraction of ASUs is distributed across the US states.

We have no direct access to other user-specific variables. Users can add additional information of themselves in their profile page, such as marital status, age, gender and number of children. This source of information is self-reported and unstructured and we were not able to extract efficiently the desired information from this source.

During the work, we explored the possibility to determine the number of children an user have and their birth date from some specific groups users follow (e.g., March 2016 Birth Club). However, we observed that it is not a good proxy.

We have added S4 Fig showing the number of users joining the forum yearly (all users and fraction of RSUs and ASUs). We observe a stable proportion of ASU joining the forum across time, around 60%. As an overall trend, we can see that the number of users joining the forum decreases starting from 2014. This may be related to the loss of popularity of the forum [3]. However, we have to keep in mind that our users correspond to the subset of the users of the forum who comment under posts related to vaccination. Thus, it is not straightforward to assess whether this trend is linked to a loss of popularity of the forum or to a loss of interest of users toward vaccination.

Fig2: instead of looking at each group separately (as it is a bit hard to make sense of all of the OR with only the name of the groups), would it be possible to create categories of groups here (e.g., "natural and healthy lifestyle" would include users in at least one of the following group: Home Birth, Organic Mamas..)?

Our analysis returned 46 out of a total of 8757 groups which are more likely to be joined by RSUs or RSUs. Creating categories of all groups, even though feasible in principle, would require to manually assign one category for each of the 8757 groups (after creating a set of categories that are suitable for these groups). We believe such a time consuming task would not add much information to the final result, as the name of the single groups are quite descriptive. However, in order to make Figure 2 more readable, we color coded groups depending on the categories already mentioned in text (vaccine skepticism, natural/healthy lifestyle, etc.).

network of users interactions: how does the network adjust for the different distributions of comments among RSU and ASU, as it seems that ASU have higher number of comments that RSU overall?

We acknowledge that the description of the interaction network may have been unclear and we thank the Referee for this observation. The interaction network is directed and weighted: the link from user i to j has weight w if there are w comments of user i under the posts of user j. This allowed us to take into account how frequently an user interact with another user from the same or the other group, and to weight accordingly the average leaning of in- and out-neighbors.

We have added information on how the interaction network was built in the Materials and Methods section: *"We represented the interactions among users as a network, where each node represents a user and there is a link from user* i *to* j *if* i *comments on a post by* j*. The link is directed and weighted, with weight corresponding to the number of comments of user* i *under the posts of user* j*. We quantified the homophily in the interactions by assigning each user a leaning with respect to scheduling ($+1$ for RSUs, -1 for ASUs), and computing for each user the average leaning of their, weighted accordingly to the weight of the links."* (Revised manuscript: lines 168:175)

l254 to 258: should be in the results section?

We have added the number of RSUs+ASUs in the Results section:
"We determined the vaccination schedule behavior of 17,015 users, 59.54% of them following an alternative vaccination schedule." (Revised manuscript: lines 195:196)

could you use your method to identify comments related to specific diseases, such as measles and pertussis,

as mentioned in the introduction? If yes, could we identify for example the temporal dynamics of the number of comments about measles by ASU and RSU and look if the number of comments increase in the last years (with the raise of cases in the US)?

In principle it can be possible to develop and extraction pipeline able to identify comments related to specific diseases. The approach would be similar to the one used to develop the extraction pipelines and it would require intensive work, particularly to find appropriate syntactic patterns and specific keywords identifying mentions of specific diseases. In addition, an evaluation of the final result would be needed and this requires manual annotation of comment. Even though it is an interesting direction of investigation, it is out of the scope of the present work.

References

[1] Babycenter. Company Information; 2020. Available from: https://www.babycenter.com/about.

[2] BabyCenter. About BabyCenter, L.L.C.; 2019. Available from: `https://www.babycenter.com/about#subtopic-bc20aboutcmpabtus`.

[3] Wexler A, Davoudi A, Weissenbacher D, Choi R, Oconnor K, Cummings H, et al. Pregnancy and health in the age of the Internet: A content analysis of online "birth club" forums. PLOS ONE. 2020;15:e0230947. doi:10.1371/journal.pone.0230947.

[4] BabyCenter. Terms of Use; 2020. Available from: `https://www.babycenter.com/help-aboutus-terms`.

[5] Fadda M, Allam A, Schulz PJ. Arguments and sources on Italian online forums on childhood vaccinations: Results of a content analysis. Vaccine. 2015;33(51):7152–7159. doi:https://doi.org/10.1016/j.vaccine.2015.11.007.

[6] Tangherlini TR, Roychowdhury V, Glenn B, Crespi CM, Bandari R, Wadia A, et al. "Mommy Blogs" and the Vaccination Exemption Narrative: Results From A Machine-Learning Approach for Story Aggregation on Parenting Social Media Sites. JMIR Public Health Surveill. 2016;2(2):e166. doi:10.2196/publichealth.6586.

[7] Lama Y, Hu D, Jamison A, Quinn SC, Broniatowski DA. Characterizing Trends in Human Papillomavirus Vaccine Discourse on Reddit (2007-2015): An Observational Study. JMIR Public Health Surveill. 2019;5(1):e12480. doi:10.2196/12480.

[8] Hample JM. Analysis of arguments favoring vaccine resistance. In: Goodwin J, editor. Between scientists & citizens: Proceedings of a conference at Iowa State University; 2012. p. 183–193.

[9] Milne A, Weijs CA, Haines-Saah RJ, McLaren L. Parents' online discussions about children's dental caries: A critical content analysis. Canadian Journal of Public Health. 2017;108(3):e265–e272. doi:10.17269/CJPH.108.5944.
