# Supplementary material for: Detecting adherence to the recommended childhood vaccination schedule from user-generated content in a US parenting forum
Source: PLoS Comput Biol. 2021 Apr 26;17(4):e1008919. doi: 10.1371/journal.pcbi.1008919 (PMC8075195; doi:10.1371/journal.pcbi.1008919)
Supplement: Attachment — Submitted filename: Response_to_reviewers.pdf [file pcbi.1008919.s009.pdf]

1. Replies to the comments of the First Referee

Thank you for the opportunity to review the revised manuscript, which has improved substantially from the original submission. I have been through the responses and the changes are generally appropriate, and below I have made comments only on the changes that were incomplete or may be inadequate and may remain as issues in this revised manuscript.

On "audience" (comments and responses were not numbered), it is worth looking through the work of Bode and Vraga (and cited/citing work) in the last three years, who have worked on several experiments related to the effect on the "audience" for interventions on social media.

We thank the Referee for having pointed out these works of which we weren't aware of. The results of these experiments may be of great value to inform policy makers on the impact that different intervention strategies would have on the audience. We have added the references to two of these works in the Discussion section to emphasize that there is evidence also on the positive effect different digital intervention strategies may have to the audience.
"Already, there is evidence showing that interventions based on websites combining vaccine information and interactive social media components can influence positively the behavior of parents [1]. Similarly, other experiments show the ability to correct health-related misconceptions also of users who do not directly participate in the discussion on social media (a phenomenon called "observational correction") [2, 3]." (Revised manuscript: lines 360:362)

The comment on the release of data and the use of public data seem to be in conflict. If the data are exempt from IRB approval (which I don't believe was sought) because they are in the public domain and can be accessed, then why can't the data also be made available so that readers can independently assess the robustness of the claims made in the work? Essentially this is saying: "There were no ethical requirements for not sharing the data and the data are public, but we won't provide the snapshot of data we collected from the website with readers to allow them to verify our claims." Either there are ethical reasons why the data can't be shared or the data should be shared.

We confirm that data are publicly available on the forum hosted by Babycenter.com and can be collected following the procedure described in the paper. However, at the same time, the Terms of Use of the website explicitly prohibits redistribution of materials made available on the platform (`https://www.babycenter.com/help-aboutus-terms`), as stated in the Data availability statement in the manuscript. Thus, even if ethical approval to collect and analyze these data is not required, we believe we have to be respectful of the Terms of Use of the platform data which prevents us from re-sharing them outside the platform itself.

The Second Reviewer's comment on overfitting was not addressed.

With respect to overfitting, the Second Reviewer asked if we used manually labeled comments both as validation and held-out test set, since in this case the performance scores would be overestimated. We answered that the labeled comments were used only to evaluate the pipelines and all the developmental steps were performed without looking at these labels. This makes the developmental steps independent of the labeled comments. We also agreed with the Second Reviewer on the overfitting issues of rule-based algorithms and the possibility that our pipelines may hardly generalise to other domains.

In order to further clarify this point, we have added a sentence in the Materials and Methods section:
"This set of manually labeled comments were used as test set." (Revised manuscript: line 100). We hope this helps to definitively clarify this important point raised by reviewers.

2. Replies to the comments of the Second Referee

The authors have addressed all my comments.

We thank the Referee for the constructive comments provided.

3. Replies to the comments of the Third Referee

I believe the authors addressed a substantial amount of my comments to a satisfactory level and I do not have any additional comments.

There are still a couple of typos:

- *l213-214: ranges from.. to*
- *l242: should be S5Fig and not S2Fig*

We thank the Referee for noticing these typos, which we fixed.

References

[1] Glanz JM, Wagner NM, Narwaney KJ, Kraus CR, Shoup JA, Xu S, et al. Web-based Social Media Intervention to Increase Vaccine Acceptance: A Randomized Controlled Trial. Pediatrics. 2017;140(6). doi:10.1542/peds.2017-1117.

[2] Vraga EK, Bode L. Correction as a Solution for Health Misinformation on Social Media. American Journal of Public Health. 2020;110(S3):S278–S280. doi:10.2105/AJPH.2020.305916.

[3] Tully M, Bode L, Vraga EK. Mobilizing Users: Does Exposure to Misinformation and Its Correction Affect Users Responses to a Health Misinformation Post? Social Media + Society. 2020;6(4):2056305120978377. doi:10.1177/2056305120978377.
